# Prognostic value of first pass stress perfusion cardiac magnetic resonance in stable coronary artery disease

**DOI:** 10.1186/1532-429X-13-S1-P114

**Published:** 2011-02-02

**Authors:** Oronzo Catalano, Guido Moro, Mauro Frascaroli, Monica Ceresa, Mariarosa Perotti, Silvia G Priori, Maurizia Baldi

**Affiliations:** 1 Fondazione Salvatore Maugeri, Pavia, Italy

## Background

Stress perfusion (SP) abnormalities predict prognosis in specific subsets of patients with coronary artery disease (CAD). However, results are more controversial in populations with stable CAD. We tried to assess prognostic value of SP cardiac magnetic resonance (CMR) in unselected patients with chronic CAD, beyond conventional risk factors such as left ventricle ejection fraction (LVEF).

## Methods

We considered consecutive patients undergoing CMR for definite/suspected chronic CAD. CMR included first-pass myocardial perfusion assessment after dipyridamole stress (0.56 mg/kg) and late gadolinium enhancement (LGE). CMR images were semi-quantitatively evaluated by two blinded operators. SP-CMR was judged positive if a perfusion defect ≥ 75% of myocardium wall thickness was detected in areas of myocardium without LGE. We also collected clinical and pharmacological history, atherosclerotic risk factors, ECG end Echocardiography data. Events in the follow-up (deaths, revascularizations > 1 month after CMR), were recorded with outpatient/inpatient visits or with structured telephonic interviews. Univariate and multivariate Cox hazard analyses were performed, with continuous variables categorized according to cut-offs from the literature.

## Results

We recruited 374 patients (78% males; 64±11y), between January 2002 and December 2006. During a follow-up period of 38±21 months there were 30 deaths (8%). Moreover, 79 patients (21%) underwent revascularization procedures. Univariate analysis showed an association between SP-CMR and mortality (unadjusted HR 4.7 [1.7-12.9], p=0.009). Stronger predictors of death were LVEF (19.1 [6.5-55.7]), LVWMSI (13.2 [4.6-38.2]), LVESV (13.1 [4.9-35.3]), LVEDV (9.2 [3.5-24.6]), LGE (8.3 [3.5-19.5]), anticoagulant (6.3 [2.7-14.3]), QTc interval (5.9 [2.9-12.2]) and loop diuretics (5.5 [2.4-12.4, p<0.001 for all of them). A significant univariate association was also found with LV diastolic function (4.4 [1.9-9.9]), aldosterone antagonist (4.2 [1.9-9.0]) and QRS duration (3.8 [1.8-7.9]). At multivariate analysis SP-CMR was found to be no more significant and independent predictors of death were only LVEF (adjusted HR 8.5 [2.6-27.8], p<0.001), QTc interval (3.2 [1.5-6.8], p=0.003), non-sinusal rhythm (6.1 [1.7-22.3], p=0.006) and LGE (2.9 [1.2, 7.4], p=0.024).

A positive SP-CMR was the strongest predictor of non-CMR related revascularization procedures, at univariate as well as at multivariate analysis (adjusted HR 3.6 [2.3-5.7], p<0.001). Other independent predictors of revascularization need in the follow-up were only diabetes (2.5 [1.5-4.1], p=0.001) and smoking habit (1.8 [1.1-3.0], p=0.015).

## Conclusions

Our study showed lack of independent association between stress induced perfusion defects at CMR and mortality in a large group of unselected patients with stable CAD. Conversely, stress CMR was the strongest predictor of subsequent revascularization procedures unrelated to CMR itself.

